# Functional Characterization of cis-Elements Conferring Vascular Vein Expression of At4g34880 Amidase Family Protein Gene in Arabidopsis

**DOI:** 10.1371/journal.pone.0067562

**Published:** 2013-07-02

**Authors:** Xuelong Wu, Ruizhi Huang, Zhihong Liu, Guoping Zhang

**Affiliations:** 1 Department of Agronomy, Key Laboratory of Crop Germplasm Resource, College of Agriculture and Biotechnology, Zhejiang University, Hangzhou, Zhejiang, China; 2 Institute of Virology and Biotechnology, Key Laboratory of Plant Metabolic Engineering of Zhejiang Province, Zhejiang Academy of Agricultural Sciences, Hangzhou, Zhejiang, China; Florida State University, United States of America

## Abstract

The expression of At4g34880 gene encoding amidase in Arabidopsis was characterized in this study. A promoter region of 1.5 kb on the upstream of the start codon of the gene (referred as AmidP) was fused with *uidA* (GUS) reporter gene, and transformed into Arabidopsis plant for determining its spatial expression. The results indicated that AmidP drived GUS expression in vascular system, predominately in leaves. Truncation analysis of AmidP demonstrated that VASCULAR VEIN ELEMENT (*VVE*) motif with a region of 176 bp sequence (−1500 to −1324) was necessary and sufficient to direct the vascular vein specific GUS expression in the transgenic plant. Tandem copy of VVE increased vascular system expression, and 5′- and 3′- deletions of VVE motif in combination with a truncated −65 CaMV 35S minimal promoter showed that 11bp cis-acting element, naming DOF2 domain, played an essential role for the vascular vein specific expression. Meanwhile, it was also observed that the other cis-acting elements among the VVE region are also associated with specificity or strength of GUS activities in vascular system.

## Introduction

Water, nutrients and signaling molecules are fundamental chemicals, which paly their specific pivotal roles in plant growth, and they are transported through a continuous vascular system within a plant. The pattern of vascular vein formation in a leaf is quite fascinated for developmental biologists because of its central importance for plant performance [1]. Actually there is a large variation among plant species, with a net-like network in dicots (reticulate venation) and a paralleling pattern in monocots (parallel venation). In plants, the two-dimensional vascular network of leaves is a paradigm of tissue formation [2]. Although the underlying molecular mechanism is poorly understood but it is believed that there exists a commonly basic mechanism during the spatial arrangement. Accumulating evidences indicate that the polar transport of phytohormone auxin plays a major and important role in vascular strand formation and differentiation [3]. It is well documented that the basipetal transport of auxin from the leaf margin, which is thought to be an essential source of auxin in the developing leaves, may control the venation pattern [4]. Not surprisingly, mutants defective in auxin perception or polar auxin transport are associated and linked with the disruption of leaf venation [3], [4].

The vascular system in the leaves of a higher vascular plant, commonly known as leaf veins or venation, is a kind of vascular bundle, mainly consisting of xylem and phloem tissues. It is well known that in plants water and minerals are transferred upward through xylem, while photo-assimilates are exported from source leaves into green and non-green sink organs through phloem [5]. The gene expression in leaf veins is usually regulated by the alteration of environments and physiological metabolic signals of this tissue during leaf development and growth. Additionally, xylem and phloem contain lots of morphologically and functionally different cell types. Therefore, it remains a challenge to obtain expression profiles of the genes specific to vascular tissue, especially those involved in transport functions [6]. Various approaches and strategies have been used to identify candidate genes, such as enhancer trap [7], mRNA from phloem cells by laser capture micro-dissection [8], fluorescence labeled cell sorting [9], and profiling expression patterns of *in vitro* differentiating vascular cells [5]. A gene encoding cinnamoyl-coA reductase from *Eucalyptus gunnii* (*EgCCR*), which catalyzes the first step in the biosynthesis of the monomeric units of lignins, by using *EgCCR* promoter analysis and *in situ* hybridization, was proved to be specific to vascular tissues [10]. In Arabidopsis, a sucrose-H^+^ symporter gene *AtSUC2* was identified by promoter analysis [11]. *AtSUC2* gene belongs to the major facilitator superfamily (MFS), including hexose transporters [12], [13]. Furthermore by analysis of ^14^C-labelled sucrose, it was found that expression of *AtSUC2* was source-stage dependent and initiated in sucrose loading, verifying that *AtSUC2* is an ideal marker gene for the sink-source transition in leaves [14]. A 126 bp fragment of the *AtSUC2* promoter is sufficient to direct the companion-cell (CC) specific and source leaf specific expression of the reporter gene [15]. Interestingly, *AtSUC2* in minor and major veins is activated by different regulatory parameters [14]. By screening an enhancer trap library, MATURE MINOR VEIN ELEMENT1 (*MMVE1*) was identified as the first minor vein specific element [7]. However, each individual strategy has its own specific limitation in determining vascular expression genes or promoters, suggesting and encouraging new approaches or all those new findings that are contributing towards more complete compendia of vascular expressed genes.

Amidases are ubiquitous enzymes and their biological functions vary greatly as they can hydrolyse wide variety of amides including arylamides, α-aminoamides, and α-hydroxyamides [16]. However, little information about amidase in plant tissues is available yet [17]. In the model Arabidopsis plants, seven so-called amidase signature (AS) members, each encoded by a different gene constitute the small enzyme family, of which only two members, AMI1 and FAAH, were studied for their enzymatic activities [18]. AMI1 is considered to function in *de novo* indole-3-acetic acid (IAA) synthesis in Arabidopsis [17], [19]. In this research work, data on the tissue-specific expression of the promoter from the amidase gene (At4g34880) are presented. Using a translational fusion of amidase promoter (AmidP) to the *GUS* reporter gene, the expression pattern of AmidP-*GUS* was studied. The results derived, demonstrated that AmidP directs a pattern of VASCULAR VEIN EXPRESSION (*VVE*) of GUS activity in *A. thaliana*. A 176 bp *VVE* motif was also identified and verified to be sufficient to drive vascular specific expression of the reporter gene. Moreover, deletion analysis indicated a DOF2 cis-element as a major regulatory element in the *VVE* motif.

## Materials and Methods

### Growth Conditions and Plant Transformation

*Escherichia coli* strain DH5α was used in the gene cloning. The final vectors were introduced into the *Agrobactium tumefaciens* strain GV3101. Transformed *A. tumefaciens* strains were introduced into *A. thaliana* wild type Col-0 plants through floral dipping [20]. Seeds from the treated plants were collected and screened for basta resistance and then the resistant plants were detected by PCR. Wild type Col-0 and transgenic plants were grown in potting soil or on half-strength Murashige–Skoog (1/2 MS) medium under 120 µmol m^−2^ s^−1^ light in a growth room having temperature of 22°C to 24°C under 16 h light/8 h dark regime and having 65% of relative humidity. Self-fertilization was allowed for the identified transgenic plants and also for the control plants, and the resulting progenies were planted for further use in subsequent experiments.

### Construction of AmidP-GUS Fusion Vector

Amidase gene (At4g34880) was isolated from Arabidopsis genomic DNA by PCR using primers AmidP1Eif and AmidPNiR (Table 1). The PCR product was subsequently ligated to pGEM-Ti vector (Promega). The obtained sequences of AmidP were confirmed by restriction analysis and nucleotide sequencing at Sangon company (Shanghai Sangon). The full 1.5 kb AmidP promoter was introduced in frame in front of *GUS* gene of pFGC-DR, a binary vector containing the *GUS* reporter gene [21], thus forming the resulting AmidP-*GUS* construction, designated as P1-DR. The P1-DR was verified by restriction analysis and subsequently nucleotide sequencing.

**Table 1:** Various primers used in this experiment.

primer name	sequence (5′–3′)	Primer pair and purpose
AmidP1EiF	GAATTCGGATAACGAGTGTTGTGGCA	AmidP1EiF/AmidPNiR for amplication of the 1.5 kb AmidP; also used to amplify the *VVE* motif with Amid-P1320Bir or deletion fragments
AmidPNiR	AACGCAGAGCAACCATGGCTAGAG	
AmidP2EiF	GAATTCCACGCGACTTCAACCCTAG	In combination with AmidPNiR to P2 fragment PCR
AmidP4EiF	GAATTCATGTGTCAAGGAGGTGA	In combination with AmidPNiR to P4 fragment PCR
AmidP5EiF	TAGAATTCTGAGTGAGAAAGTGAGAGACT	In combination with AmidPNiR to P5 fragment PCR
AmidP6EiF	GAATTCTTTGTTACACGCAAATCTG	In combination with AmidPNiR to P6 fragment PCR
GUS2EiBif	ACTGAATTCTCACAGGATCCGCAAGACCCTTCCTCT	GUS2EiBif/GUSXiR for amplication of the -65
GUSXiR	AATTCTAGAGCTGGTCACCTGTAAT	minimal 35S promoter and GUS gene
Amid-P1500BIIf	ACTAGATCTGGATAACGAGTGTTGTG	Amid-P1500BIIf/Amid-P1320Bir for amplication of the *VVE* motif
Amid-P1320Bir	AGTGGATCCTAGGCTCCTTTCC	In combination with AmidPNiR to P2 fragment PCR
5M1-160Eif	TCAGAATTCGTGGCAAAAAAATGCTC	Used to construct the 5M1 deletion fusion with Amid-P1320Bir
5M2-140Eif	TCAGAATTCATACACCAAAAAATTG	Used to construct the 5M2 deletion fusion with Amid-P1320Bir
5M3-120Eif	TCAGAATTCTGTTAAAATAAGATATTGG	Used to construct the 5M3 deletion fusion with Amid-P1320Bir
5M4-100Eif	TCAGAATTCTTTAGGCTTTTGGAG	Used to construct the 5M4 deletion fusion with Amid-P1320Bir
3M1-160Bir	AGTGGATCCTAATTGATTTGATTCTT	Used to construct the 3M1 deletion fusion with AmidP1EiF
3M2-140Bir	AGTGGATCCAGATGGACCGACAGA	Used to construct the 3M2 deletion fusion with AmidP1EiF
3M3-120Bir	AGTGGATCCGGCGTTGGAGTTTAGG	Used to construct the 3M3 deletion fusion with AmidP1EiF
3M4-100Bir	AGTGGATCCGACTCCAAAAGCCTAAA	Used to construct the 3M4 deletion fusion with AmidP1EiF
3M5-90Bir	AGTGGATCCAAGCCTAAAGCCCAATATC	Used to construct the 3M5 deletion fusion with AmidP1EiF
FGCf2	CGGATACTTACGTCACGTCTTGC	Used to PCR verification of the final constructs with GUS45r
GUS45r	GAGAAAAGGGTCCTAACCAAGA	

### Construction of Truncated AmidP-GUS Fusions

A series of 5′-flanking region truncations of the AmidP (naming P2, P4, P5, and P6 with length of 1324 bp, 902 bp, 581 bp, and 300 bp nucleotides, respectively) were obtained by PCR with the 1.5 kb AmidP as a template and by using the same reverse primer AmidPNiR and different forward primers, nearly 300 to 400 bp subtractions in length were obtained (Table 1). The truncated fragments of AmidP promoter were inserted in front of *GUS* gene of pFGC-DR to construct vectors of P2-DR, P4-DR, P5-DR, and P6-DR, respectively. A P3-DR vector containing the initial 176 bp sequences but without sequences between P2 and P5 fragments was obtained by self-ligation of the P1-DR plasmid, digested by *Bam*HI and *Bgl*II within the 1.5 kb AmidP. The P3 fragment comprised 805 bp sequences including 4 nucleotides arbitrarily added to form a *EcoR*I site on the 5′ flanking. All the truncated AmidP fragments *GUS* fusions were further verified and sequenced.

### Generation of Fusions Containing Tandem Copies of the VVE Motif and −65 Minimal 35S-GUS Cassette

In order to generate the fusion constructions containing tandem copies of the *VVE* motif and CaMV 35S minimal promoter, a fragment of −65 minimal 35S and *GUS* gene amplified from the plasmid pCambia1301 by PCR with primers GUS2EiBif and GUSXiR (Table 1) was cut with *EcoR* I and *Xba* I, and then inserted into the same enzymes cutting pFGC5941 to produce an intermediate pFGC-MiniGUS construction, which contained a −65 minimal 35S promoter at the upstream of the coding region for *GUS*. To generate the tandem copies of *VVE*, the 176 bp *VVE* motif was amplified from the AmidP plasmid with primers AmidP1EiF and Amid-P1320Bir or Amid-P1500BIIf and Amid-P1320Bir (Table 1), respectively. Three fragments of *EcoR*I and *BamH*I cutting *VVE*, *Bgl*II and *BamH*I cutting *VVE*, and *EcoR*I and *BamH*I cutting pFGC-MiniGUS were ligated in a reaction. Clones were then determined by the orientation and number of *VVE* inserts. Thus, three constructs named VVE, 2VVE-1, and 2VVE-2 were obtained, they contained one copy, two copies in the same orientation and two copies in reverse orientation of the *VVE* motif, respectively.

### Generation of Fusions Containing 5′ and 3′ Deletion of the VVE Motif and −65 Minimal 35S-GUS Cassette

In order to define which cis-element in the *VVE* motif is essential for the vascular vein expression, fine 5′ and 3′ deletions of the *VVE* motif were performed and combined with the −65 minimal 35S promoter to drive *GUS* reporter gene. The deletions were all carried out by PCR amplification with respective primers (Table 1). Fragments of *EcoR*I and *BamH*I cutting PCR products were inserted into the same enzymes cutting pFGC-MiniGUS to produce the 5′ and 3′ deletion fusions, namely 5M1 to 5M4 and 3M1 to 3M5, respectively. Every deletion was 20 nucleotides excised except the 5^th^ 3′ deletion which was 10 nucleotides excised.

### Histochemical and Fluorometric GUS Assays

For *GUS* histochemical staining, ten-day seedlings grown in potting soil or on 1/2 MS medium or tissues from 40 d mature plants were immersed in the *GUS* staining buffer (50 mM sodium phosphate buffer, pH 7.0, 1 mM EDTA, 0.5 mM potassium ferricyanide, 0.5 mM potassium ferrocyanide, 0.1% Triton X-100, 1 mM X-gluc) at 37°C for 4 h to 12 h. The reaction was stopped by repeated rinsing in ethanol. For the quantitative analysis, a single leaf from the independent T1 lines of each tandem construction was stained with X-gluc. Only the plants showing *GUS* staining were included in subsequently quantitative *GUS* analysis by fluorometric quantification of 4-methylumbelliferone produced from 4-methylumbelliferyl β-D-glucuronide (MUG) [22]. Leaves of 10 d seedlings of independent transgenic lines were ground in liquid nitrogen, and soluble proteins were homogenized in the *GUS* assay buffer (50 mM potassium phosphate, 10 mM EDTA, 0.1% Triton X-100, 0.1% SDS and 0.1% 2-mercaptoethanol), and an aliquot of the supernatant was incubated in the buffer with MUG as substrate at 37°C for 2 h, then was stopped by adding 800 ml 125 mM Na2CO3. The amount of 4-methylumbeliferone formed by the *GUS* reaction was determined at OD415 using a fluorescence spectrophotometer. Protein concentration was determined using BSA as the standard. GUS activity in pFGC-DR transgenic seedlings was assigned as 100%. Each *GUS* assay was performed in triplicate from each collection of seedlings from independent transgenic lines per construction.

### In-silico Analysis

In-silico analysis of the *VVE* motif was performed using AthaMap web tool (http://www.athamap.de; Bulow et al., 2006).

### Accession

Sequence of the AmidP can be found in the GenBank data libraries under accession number NC_003075.7. Sequence of the pFGC5941 plasmid is provided at GenBank under accession number AY310901.

## Results

### The AmidP Drives Expression in Vascular Vein Expression

**Figure 1:**
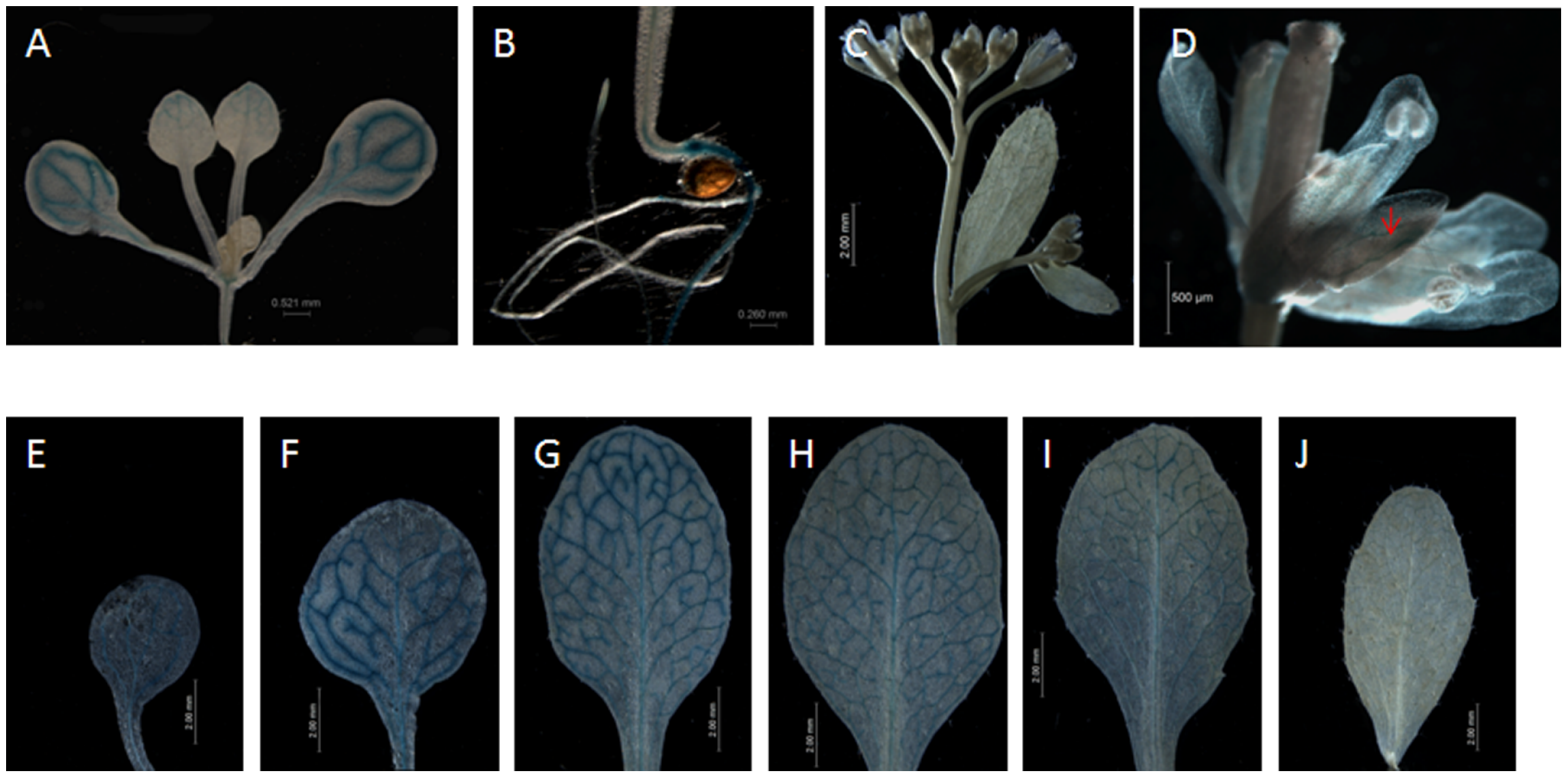
The AmidP drives the GUS expression in the vascular vein of leaves resembling a pattern of the sink-to-source transition. A. GUS expression is detected in cotyledons and the distal tip of young leaves of 10-d seedlings. B. The AmidP drives expression in the germinating seed joint of above- and under-ground part. C–D. GUS activity is detected in sepals of flowers (C), as shown in an amplified flower indicated with a red arrow (D). E–J. X-Gluc staining is detected throughout the vascular veins of a cotyledon (E), expanded source leaves (F, G, and H) and progresses basipetally down transition leaves (I and J).

To determine the tissue-specific expression of the At4g34880 gene encoding amidase, a translational fusion named P1-DR containing the AmidP with the sequences of 1.5 kb nucleotides and a *GUS* gene in frame was transformed into Arabidopsis. AmidP-*GUS* expression was clearly observed in the vascular tissue of cotyledons, the distal tip of young leaves, and in the germinating seed joint of above and under-ground part of 10 d seedlings. The *GUS* localization at the vascular veins in 10-d cotyledons was readily detectable (Figure 1A and 1B). Weak *GUS* activities were also observed in the vascular veins of sepals (Figure 1C and 1D). To detect the *VVE* pattern of the *amidase* gene in leaves, rosette leaves from 40-d P1-DR transgenic plants were subjected to *GUS* histochemical staining. X-gluc staining was restricted to the vascular veins of cotyledons (Figure 1E), expanded source leaves (Figure 1F to 1I) and basipetally down transition leaves (Figure 1J), resembling a pattern of sink-to-source transition. These expression patterns are similar to those observed in *AtSUC2* gene [11].

### Truncation Analysis of the AmidP

**Figure 2:**
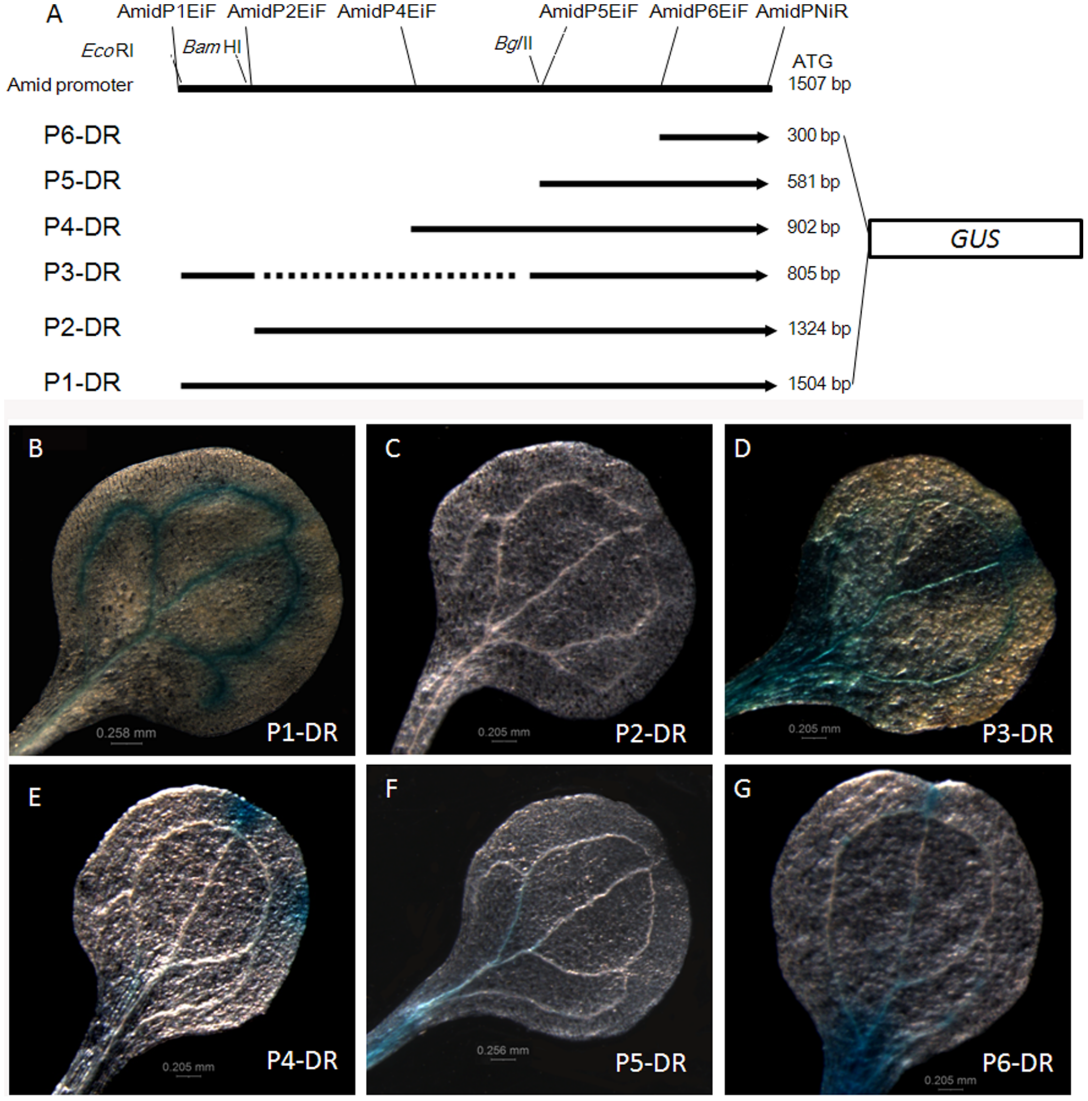
Truncation analysis of the AmidP in cotyledons of transgenic Arabidopsis seedlings. A. Promoter truncation, the primers binding sites and endonucleases sites are illustrated. The 1504 bp cloned sequence for the AmidP includes 4 nucleotides which are added to form an *Eco*RI acting site. All the fused structures are obtained by ligation of the pFGC-DR and the PCR products precut by *Eco*RI and *Nco*I respectively, except that of the P3-DR which is self-ligated with the P1-DR digested by *Bam*HI and *Bgl*II. B-G. GUS activities in the transgenic Arabidopsis cotyledons of P1-DR (B), P2-DR (C), P3-DR (D), P4-DR (E), P5-DR (F), and P6-DR (G).

AmidP activities in leaves were restricted to source leaves and to the source areas of transition leaves. To further define the cis-elements involved in the regulation of this expression pattern within 1.5 kb sequences of AmidP, a series of truncated promoter with *GUS* fusion named as P2-DR, P3-DR, P4-DR, P5-DR, and P6-DR were produced based on the P1-DR vector (Figure 2A). All those truncated promoter vectors were introduced into Arabidopsis for analysis of *GUS* expression. At least five independently transgenic plants were used to characterize the activity of each truncated promoter. In the experiments, the pattern of *GUS* activity in cotyledons for each fusion vector was usually similar to that in other mature source leaves. So the expression pattern for each vector of *GUS* activity in cotyledons was characterized and was used for further analysis. *GUS* staining was restricted to vascular vein in all plants expressing longest P1-DR construct, while in contrast the *GUS* activities were abolished in the plants harboring the P2-DR construction, which was excised from a region of 176 bp sequences on the 5′ upstream of the 1.5 kb AmidP (Figure 2B and 2C). The differences of *GUS* activities between P1-DR and P2-DR transformed plants demonstrated that the 176 bp region in the 5′ upstream of AmidP is sufficient to drive a pattern of vascular vein expression of *GUS* reporter gene. Thus, the 176 bp region was designated as *VVE* (*vascular vein expression*) motif. The role of *VVE* motif for the specific pattern of *GUS* expression was also observed in transgenic plants harboring P3-DR and other three constructions (P4-DR, P5-DR and P6-DR) with or without the *VVE* motif, respectively. *GUS* activities in transgenic plants harboring the P3-DR construction, which had the *VVE* motif but without a region from −1324 to −581, could be seen in the vascular veins, but were not so specific as that in P1-DR transgenic plants, with a strong *GUS* staining in regions near the petiole and apex (Figure 2D). While histochemical localizations of *GUS* activity in transgenic plants harboring the other three vectors without the *VVE* motif were much weaker and not specific in vascular veins. *GUS* activities appeared in the secretory structures of hydathodes (P4-DR), non-specific in sections of veins (P5-DR), and mainly in regions near the petiole and apex of cotyledons (P6-DR) (Figure 2E to 2G). These results demonstrated that the Amidase promoter has an important *VVE* motif for the vascular vein expression of *GUS* activities and other regions to regulate the specificity or strength of this expression pattern.

### Tandem Copies of the VVE Increase Strength of GUS Activities

**Figure 3:**
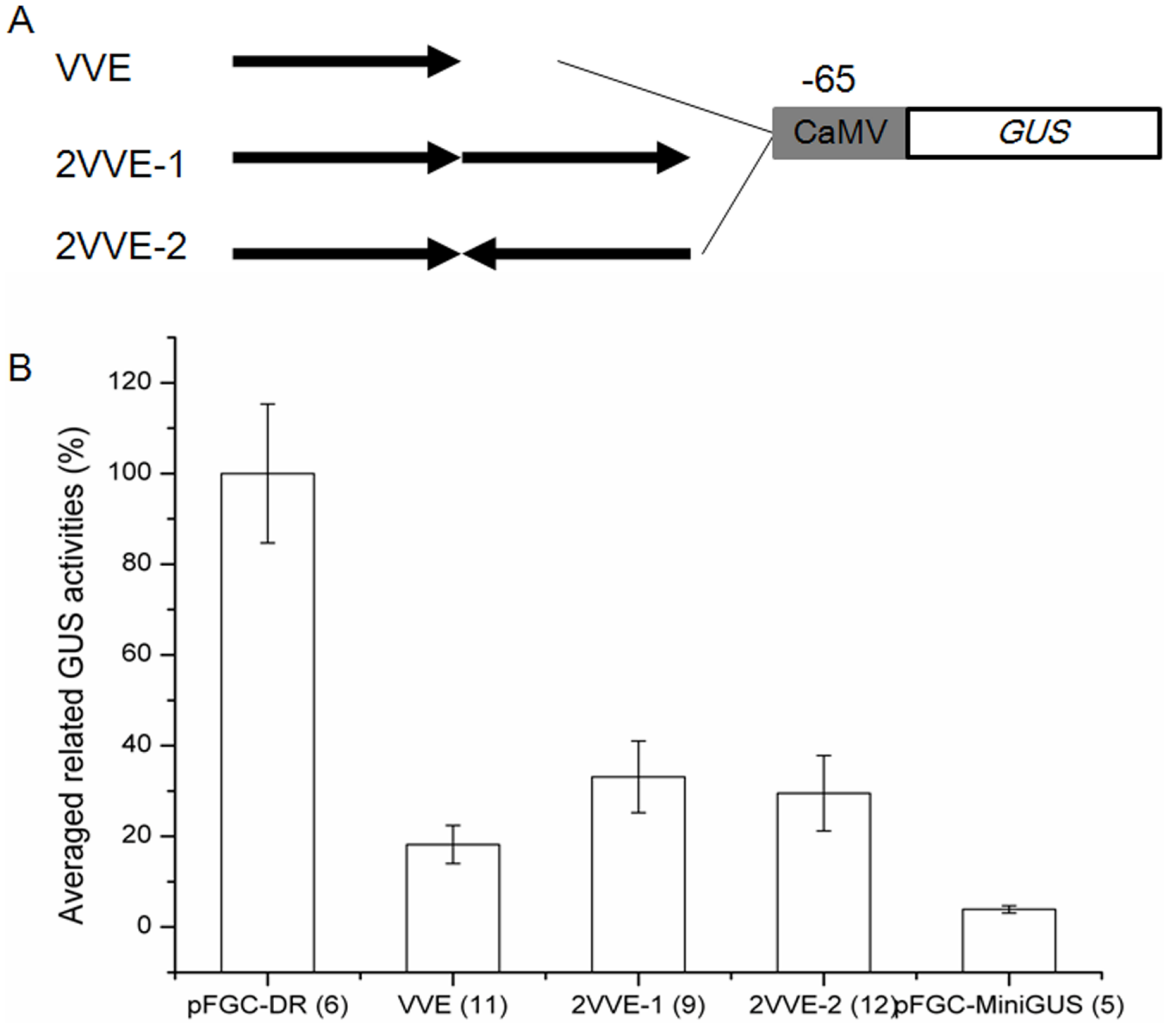
Tandem copies of *VVE* (or the reverse complement orientation) motif on the strength of GUS activities. A. The number and orientation of *VVE* tandems is illustrated (not to scale). *VVE*, represented with black arrows, is placed upstream of the minimal 35S promoter (−65; black box) to drive *uidA* (blank box) expression (see “Materials and methods”). B. Quantitative GUS activity analysis of the tandem construction in leaves of 10 d transgenic Arabidopsis seedlings. pFGC-DR and pFGC-MiniGUS were used as positive and negative controls, respectively. GUS activity in pFGC-DR transgenic seedlings was assigned as 100%. GUS activity is replicated three times of each collection of seedlings from independent transgenic lines (indicated in parentheses) per construction. Error bars are standard deviations.

To demonstrate the strength and potential application of this motif, *VVE* or tandem copies of *VVE* were used to drive the *GUS* reporter gene in combination with a CaMV 35S minimal (−65) promoter (Figure 3A). For the analysis, we used the minimal 35S promoter from the pFGC-MiniGUS plasmid containing the TATA-box region but without other functional cis-elements. The pFGC-MiniGUS transformed plants showed very weak *GUS* activities, compared to transgenic plants harboring the complete 35S promoter pFGC-DR or the VVE vectors (Figure 3B). *VVE* motif and −65 minimal 35S fusion promoter could drive *GUS* expression in an expected pattern as that of the AmidP (results not shown). As shown in Figure 3B, two copies of *VVE* motifs in either same direction or opposite direction nearly increased double strength of *GUS* expression of a *VVE* monomer, suggesting that *VVE* motif can be applied as a vascular vein expression specific enhancer.

### In-silico Analysis of the Putative Cis-element and Deletions of VVE Motif

**Figure 4:**
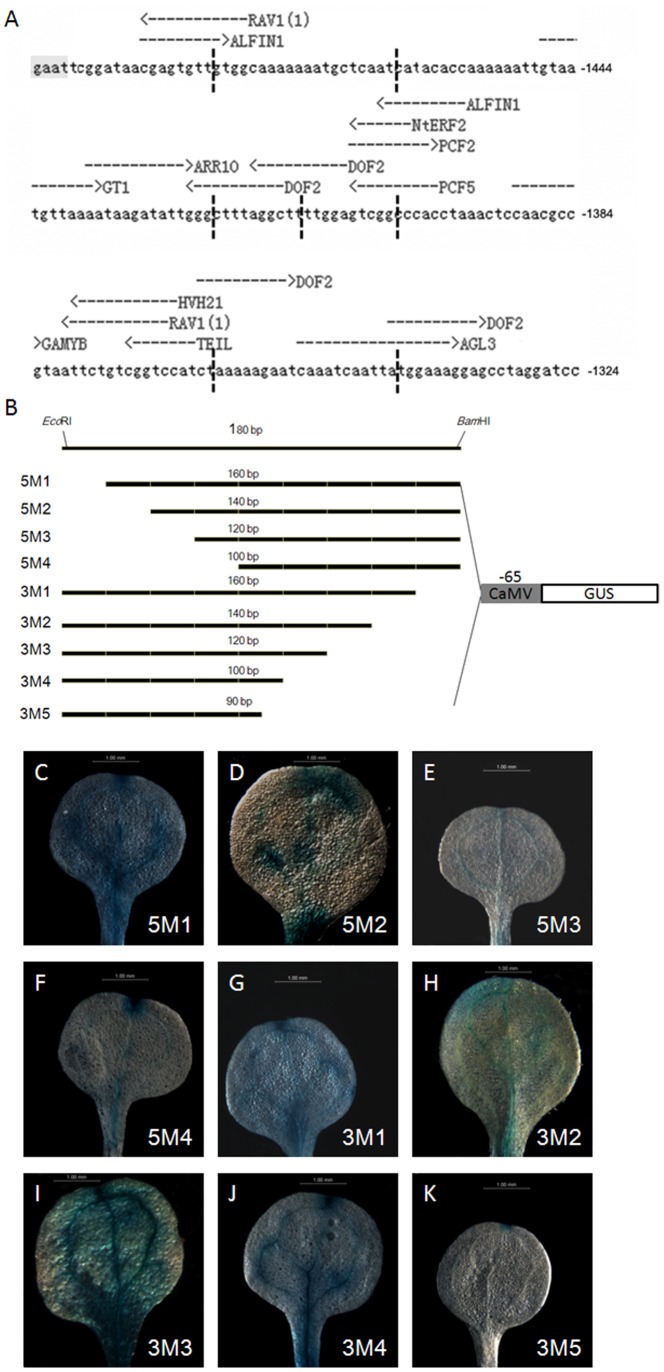
The construction and results of the 5′- and 3′- deletions of *VVE* motif between −1500 and −1324 of the AmidP. A. Sequence and element site analysis of the *VVE* motif in the AmidP. 4 nucleotides (grey box) are arbitrarily added to form an *Eco*RI acting site. Element sites for known transcription factors indicated as arrows are detected by AthaMap web tools (see “Materials and methods”). Vertical dotted line indicated deletion sites. B. Schematic diagram of the chimeric constructs. The numbers above the bars indicate the residual region of the *VVE* motif after 5′- or 3′- deletions. All the fused constructs are obtained by ligation of the pFGC-MiniGUS and the PCR products precut by *Eco*RI and *Bam*HI respectively. C–K. Representative histochemical stained cotyledon demonstrates the strength and specificity of GUS activities in the transgenic Arabidopsis of 5M1 (C), 5M2 (D), 5M3 (E), 5M4 (F), 3M1 (G), 3M2 (H), 3M3 (I), 3M4 (J), and 3M5 (K).

To determine the essential cis-elements for the specific expression pattern in *VVE* motif, 176 bp *VVE* motif was used to perform in-silico analysis of the binding sites of transcript factors using AthaMap web tool (http://www.athamap.de; Bulow et al., 2006). The predicted sites included 4 DOF2-domain, 2 ALFIN1, 2 RAV1, and some other motifs with one copy per site (Figure 4A). Based on the length of the whole *VVE* motif sequences and the results of the in-silico analysis, four 5′ and five 3′ deletions of *VVE* motif combined with the -65 minimal 35S promoter consecutively named as 5M1 to 5M4 and 3M1 to 3M5 respectively, were created to drive the *GUS* reporter gene. With 4 nucleotides arbitrarily added in 176 bp *VVE* motif, each deletion was excised 20 nucleotides except the 3M5 which was excised 10 nucleotides (Figure 4B). X-Gluc staining were performed to analyze the activities of *GUS* reporter gene with cotyledons from at least 5 independent transgenic lines for each construction, it was found that nearly each deletion could change the specificity or the strength of the vascular vein expression. 5M1 and 3M3 could increase the strength of *GUS* activities but decrease the specificity in veins (Figure 4C and 4I). In contrast, *GUS* expression of 5M3 or 5M4 was restricted in veins but the strength decreased to some degree (Figure 4E and 4F), and *GUS* activities for 5M2 and 3M2 were the greatest in veins but discontinuous (Figure 4D and 4H). The expression pattern of *GUS* gene in 3M1 or 3M4 was similar to that in P1-DR (Figure 4G and 4J). However, in cotyledons from 3M5 transgenic plants, only possible hydathode site was stained with some lines (Figure 4K). These results indicated that the second DOF2-domain disrupted by the 3M5 deletion is the possibly essential cis-element for specific expression pattern of *VVE* motif, and other elements in this *VVE* region also have effects on its specificity and strength.

## Discussion

Amidases are a kind of enzymes that are ubiquitously present in various organisms [16], [17], [23], [24]. These enzymes belong to the carbon-nitrogen hydrolase superfamily usually involved in the final reduction step of N utilization [25]. Amidase encoded by the AS family gene have the function of amidohydrolase in carbon-nitrogen hydrolyzation. The amidase family in *Arabidopsis thaliana* has been found to comprise seven putative members based on the conserved core structure of the family, all characterized by the glycine- and serine- rich AS motif [17]. However, these seven members are clustered into four subgroups. AIM1 along with mtOM64 and Toc64-III proteins forms the first group. The second group consists of FAAH and At3g25660. Two remained proteins encoded by At5g07360 and At4g34880 branch directly from the origin [17]. To date, AMI1, one of the two known members so far in the AS family, has been well documented to work in producing IAA and NAA by hydro-cleavage of indole-3-acetamide (IAM) and 1-naphthaleneacetamide (NAM), respectively [17]. While other known members FAAH can cleave substrates like oleamide and acylethanolamines to release fatty acids and ammonia [26]. These two members have amidase activities in plants. The products of the amide hydrolyzation comprised of nitrogen like amino acids that are involved in long-distance translocation and in xylem and phloem exchange. It is known that source leaves is a kind of nitrogen demanding tissues, which needs to remobilize nitrate from older sink leaves. NRT1.7 plays such a role in source leaves, remobilizing nitrate in phloem loading from older leaves in Arabidopsis [27]. Another example is the *AAT1* gene, which encodes a amino acid transporter in Arabidopsis and is specifically expressed in major veins of leaves and roots [28]. Here, AmidP from the amidase encoded At4g34880 gene drives expression of *GUS* reporter gene in vascular tissues, resembling a pattern of sink-to-source transition characterized by strong expression in source leaves like cotyledons and mature leaves, and also the source region of a transition leaf, but weak or even no expression in sink tissues (Figure 1). The pattern is very similar to that of the sink-to-source transition marker *AtSUC2* gene [11]. Usually, expression pattern of a gene by promoter analysis could reflect its function. Thus, its assumed that At4g34880 gene might function in vascular tissues in leaves during sink-to-source transition.

Due to the fact that the promoter regions differ greatly among the different genes, the assumed promoter region for promoter motif in different analysis varies largely (250 bp to 28600 bp upstream of the transcription factor start site) [29]. In addition, functional transcription factor binding sites are unevenly distributed along the promoter and are non-specific to a particular region. Truncation analysis of AmidP demonstrates that a 176 bp fragment (−1500 to −1324) existed in the 5′ flanking region is necessary for vascular expression designated as *VVE* motif (Figure 2). Additionally, there are some other regions, which negatively regulate the vascular expression. For example, transgenic plants harboring P4-DR construct demonstrated *GUS* staining in the hydathodes, a kind of secretory structures in exchanging water between the interior and surface of a leaf (Figure 2E). Tandem copies of the *VVE* motif combined with -65 minimal 35S promoter suggested that the *VVE* motif is not only responsible for the vascular vein expression, but it can also act as a enhancer by accurately measuring fluorometric *GUS* activities (Figure 3). In-silico analysis of the known transcription factor binding sites identified several cis-elements in the *VVE* motif [30]. The fine 5′ and 3′ deletions further demonstrated that the second DOF2 domain has a major role in the vascular expression of *VVE* motif (Figure 4). Other deletions have effects on the specificity or the strength of vascular expression pattern, but vascular vein expression of *GUS* reporter gene was not abolished (Figure 4). DOF are plant specific transcription factors and have been demonstrated in EST libraries from vascular tissue mRNA [31]. In 176 bp *VVE* motif, there are three DOF2 cis-elements. In fact, a DOF protein involved in controlling glucosinolate biosynthesis is phloem specifically localized [32]. Potential DOF binding sites were also found in the vascular specific promoters of *GmSBP2* gene from *Glycine max* [33], *CmGAS1* gene from *Cucumis melo* [34], and *AtSUC2* gene from *Arabidopsis thaliana* respectively [35]. Similar presence of DOF binding sites in vascular specific promoters not only indicated and expressed the important role of DOF element for the specific expression pattern but a conserved mechanism for the transcriptional regulation of these genes was also illustrated.
